# Sika deer (*Cervus nippon*)-specific real-time PCR method to detect fraudulent labelling of meat and meat products

**DOI:** 10.1038/s41598-018-25299-7

**Published:** 2018-05-08

**Authors:** Maria Kaltenbrunner, Rupert Hochegger, Margit Cichna-Markl

**Affiliations:** 1 Austrian Agency for Health and Food Safety, Institute for Food Safety Vienna, Department of Molecular Biology and Microbiology, Spargelfeldstraße 191, 1220 Vienna, Austria; 2 Department of Analytical Chemistry, Faculty of Chemistry, University of Vienna, Währinger Straße 38, 1090 Vienna, Austria

## Abstract

Since game meat is more valuable and expensive than meat from domesticated animal species it is a potential target for adulteration. Analytical methods must allow the identification and quantification of meat species to be applicable for the detection of fraudulent labelling. We developed a real-time PCR assay for the authentication of sika deer (*Cervus nippon*) and products thereof. The primer/probe system amplifies a 71 bp fragment of the *kappa-casein precursor* gene. Since the target sequence contained only one sika deer-specific base, we introduced a deliberate base mismatch in the forward primer. The real-time PCR assay did not show cross-reactivity with 19 animal and 49 plant species tested. Low cross-reactivity was observed with red deer, fallow deer, reindeer and moose. However, with a ΔCt value of ≥11.79 between sika deer and the cross-reacting species, cross-reactivity will not affect the accuracy of the method. LOD and LOQ, determined by analysing serial dilutions of a DNA extract containing 1% (w/w) sika deer DNA in pig DNA, were 0.3% and 0.5%, respectively. The accuracy was evaluated by analysing DNA mixtures and DNA isolates from meat extract mixtures and meat mixtures. In general, recoveries were in the range from 70 to 130%.

## Introduction

Commercial food products must not pose any health risks to consumers to comply with national and international food regulations^1,2^. Since foodstuffs also have to be authentic, food producers are not allowed to deliberately make false or misleading statements, e.g. on the composition, quality, geographic origin or the processing of food products. However, food adulteration has become a global issue. Studies report that meat products frequently are adulterated with respect to the species the meat originates from^3–6^. Since meat from game animals is more valuable and expensive than meat from domesticated animal species, it is a potential target for adulteration^7^.

Deer meat is appreciated for its characteristic taste and tenderness. In addition, it is characterized by a low fat content and a balanced ratio of omega-6 to omega-3 essential fatty acids. Therefore, its consumption has been associated with positive health effects on the cardiovascular system^7,8^. In Europe, roe deer (*Capreolus capreolus*) and red deer (*Cervus elaphus*) are indigenous. Sika deer (*Cervus nippon*) was introduced from East Asia to Europe and other parts of the world by anthropogenic translocation^9,10^. Sika deer and red deer differ in morphological characteristics, such as body size, shape of the antlers and pelage pattern. Genetically, both deer species are closely related. Numerous genes show sequence homology ≥99%. In contrast, the control region of the mitochondrial DNA has been reported to show a sequence divergence of 5.02%^11^.

Analytical methods must allow both the identification and quantification of meat species to be applicable to detect deer meat adulteration. Quantitative information is necessary because according to the guidelines of the Codex Alimentarius Austriacus, in “game sausages” at least 38% of the total meat content must derive from game species^12^. Currently, DNA based methods play the most important role in meat authenticity testing. We have already developed several real-time polymerase chain reaction (PCR) assays for the identification and quantification of deer meat in food products. One real-time PCR assay allows the identification and quantification of roe deer (*Capreolus capreolus*)^13^, another one the determination of the sum of red deer (*Cervus elaphus*), fallow deer (*Dama dama*) and sika deer (*Cervus nippon*)^14^. Since there is a trend of meat producers and restaurant owners to disclose the deer species to market their product as delicacy, e.g. “sika deer filet“ or “fallow deer salami”, methods are needed that allow the identification of the individual deer species. Very recently, we have developed real-time PCR assays for the identification and quantification of fallow deer^15^ and red deer^16^.

In this study, we present a real-time PCR assay for the identification and quantification of sika deer in food products. In contrast to methods targeting mitochondrial DNA^17–19^ we attempted to target a single copy gene. Due to the constant copy number, single copy genes are better suitable for obtaining accurate quantitative results than mitochondrial DNA sequences^20^.

## Results

### Design and test of primer/probe systems

We used the *kappa-casein precursor* gene, the *nanog* pseudogene and the *protein kinase C iota* gene for the design of primers and probes. These genes were selected because in the National Center for Biotechnology Information (NCBI) database the sequences were available not only for sika deer but also for some closely related species, including red deer and reindeer. With the help of the primer design software, we identified a target sequence of appropriate length in each of the three genes. However, each target sequence contained only one sika deer-specific base. We positioned the sika deer-specific base at the 3′ penultimate site of the forward primers (primer 1 fw 1, primer 2 fw 1 and primer 3 fw 1, Supplementary Table 1). Together with the respective reverse primer and probe (primer/probe systems 1a, 2a and 3a, Supplementary Table 1), the forward primers were subjected to cross-reactivity tests with DNA isolates from red deer (*Cervus elaphus*), fallow deer (*Dama dama*), roe deer (*Capreolus capreolus*), alpine ibex (*Capra ibex*), reindeer (*Rangifer tarandus*), moose (*Alces alces*) and goat (*Capra hircus*) using the QuantiTect® Multiplex PCR NoROX Kit Master Mix (Qiagen, Hilden, Germany) (Supplementary Table 2). Each of the DNA isolates was analysed in duplicate. The amplifiability of the DNA has already been checked in a previous study by using a reference real-time PCR assay targeting the myostatin gene of mammals and poultry^21^. None of the primer/probe systems was found to be specific for sika deer. Primer/probe system 1a did not allow the differentiation between sika deer, red deer and fallow deer (mean Ct value ± standard deviation (SD) 23.67 ± 0.81) (Supplementary Table 2). In addition, PCR products were obtained for moose, reindeer and roe deer (Ct values ≤ 31.44). With primer/probe system 2a, the target sequence was amplified not only for the DNA isolate from sika deer, but also for those from the other seven species tested (mean Ct value ± SD = 23.89 ± 0.88). Primer/probe system 3a, yielding a Ct value of 27.74 for sika deer, showed cross-reactivities with other deer species (red deer Ct = 27.40, fallow deer Ct = 28.05, roe deer Ct = 27.64, reindeer Ct = 31.74, moose Ct = 27.14).

**Figure 1:**
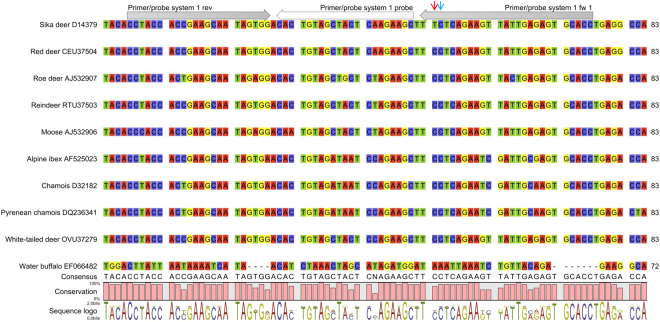
Sequence alignment of the *kappa-casein precursor* gene. Arrows indicate the position of the forward primer (1 fw 1), reverse primer (1 rev) and the probe (1 probe) of primer/probe system 1a. All forward primers of primer/probe system 1 contained the sika deer-specific base (indicated by the vertical red arrow). Forward primers 1 fw 2–4 differed from each other in the mismatch base (position indicated by the vertical blue arrow). Sequence alignment was carried out using the CLC Genomics Workbench 8.0 (Qiagen).

**Figure 2:**
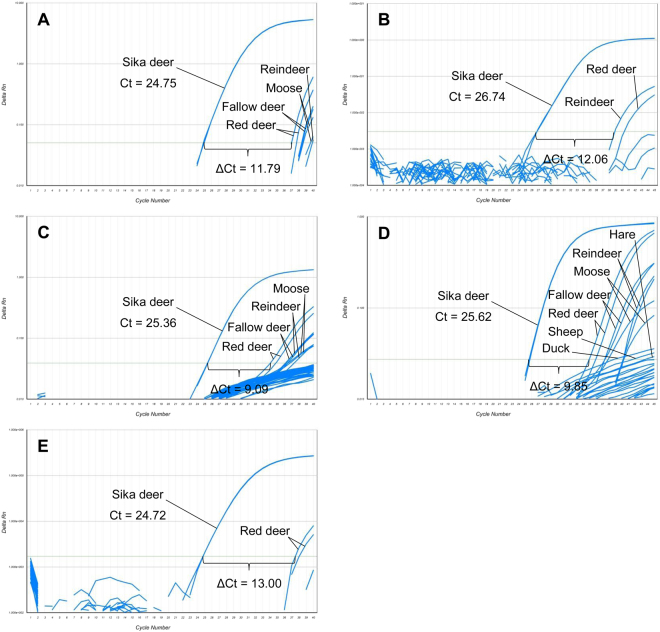
Results obtained in cross-reactivity tests performed with DNA isolates from 23 animal species with 5 commercial master mixes. (**A**) QuantiTect® Multiplex PCR NoROX Master Mix (Qiagen), (**B**) TaqMan® Universal PCR Master Mix (Applied Biosystems), (**C**) GoTaq® Probe qPCR Master Mix (Promega), (**D**) PerfeCTa® qPCR ToughMix^TM^, Low ROX^TM^ (Quanta Biosciences) and E) Takyon^TM^ No Rox Probe MasterMix dTTP Blue (Eurogentec).

To enhance the specificity for sika deer, we modified the forward primers by introducing one deliberate base mismatch in each of the primers. The mismatch was positioned adjacent to the sika deer-specific base, at the last but two site from the 5′ end (primers 1 fw 2–4, primers 2 fw 2–4 and primers 3 fw 2–4, respectively, Supplementary Table 1). The resulting nine primer/probe systems were tested for cross-reactivity with the seven animal species mentioned above (Supplementary Table 2). With primer/probe systems 2b-d and 3b-d similar results were obtained as with primer/probe systems 2a and 3a, respectively, indicating that the insert of the base mismatch did not increase the specificity for sika deer. In contrast, modification of primer 1 fw 1 turned out to be successful. Primer/probe system 1b (Supplementary Table 1, Fig. 1) yielded a Ct value of 24.75 for sika deer. Low cross-reactivity was only observed for red deer (Ct = 36.54), fallow deer (Ct = 37.71), reindeer (Ct = 38.87) and moose (Ct = 39.08) (Fig. 2A). With a ΔCt value of ≥11.79 between sika deer and the cross-reacting species, cross-reactivity will not affect the accuracy of the method. Primer/probe systems 1c and 1d (Supplementary Table 1) also showed enhanced specificity compared to primer/probe system 1a, but the ΔCt values between sika deer and the cross-reacting species were lower (≥7.49). In further cross-reactivity tests with DNA isolates from 16 animal species and 49 plant species, primer/probe system 1b did not result in an increase in the fluorescence signal within 40 cycles. Based on these results, all further experiments were performed with primer/probe system 1b.

To investigate whether the composition of the commercial master mix influenced the specificity for sika deer, we analysed DNA isolates from 24 animal species with four master mixes from other suppliers in duplicate. Three of them, the GoTaq® Probe qPCR Master Mix (Promega, Madison, Wisconsin, USA), the PerfeCTa® qPCR ToughMix^TM^, Low ROX^TM^ (Quanta Biosciences, Gaithersburg, Maryland, USA) and the Takyon^TM^ No Rox Probe MasterMix dTTP Blue (Eurogentec, Seraing, Belgium) could be used by applying fast thermal programs. The other two master mixes, the QuantiTect Multiplex PCR NoROX Master Mix (Qiagen) and the TaqMan® Universal PCR Master Mix (Applied Biosystems, Foster City, CA, USA) were used by applying conventional thermal cycling programs. The TaqMan® Universal PCR Master Mix (Applied Biosystems) led to a higher Ct value for sika deer (Ct = 26.74) than the other four master mixes (Ct ± SD = 25.11 ± 0.45), the ΔCt value between sika deer and the cross-reacting species was ≥12.06 (Fig. 2B). For the GoTaq® Probe qPCR Master Mix (Promega) (Fig. 2C) and the PerfeCTa® qPCR ToughMix^TM^, Low ROX^TM^ (Quanta Biosciences) (Fig. 2D), ΔCt values between sika deer and the cross-reacting species were ≥9.09. Best results were obtained with the Takyon^TM^ No Rox Probe MasterMix dTTP Blue (Eurogentec). The Ct value for sika deer was almost identical with that of the master mix from Qiagen (Fig. 2A), however, among the species tested only red deer led to an increase in the fluorescence signal within 40 cycles (ΔCt value 13, Fig. 2E). The thermal cycling method (fast or conventional) did not influence the Ct values obtained for sika deer and the ΔCt values between sika deer and the cross-reacting species. Thus, our sika deer-specific primer/probe system can be used with fast as well as with conventional thermal cycling programs, depending on the master mix.

### Optimisation of the primer/probe concentration

All results presented above were obtained with a constant concentration ratio of forward primer, reverse primer and probe (200 nM: 200 nM: 100 nM). Experiments performed with primer and probe concentrations up to 900 nM, and different concentration ratios indicated that both the concentration and the concentration ratio used for the cross-reactivity tests were optimal. The other primer/probe conditions led either to higher Ct values for sika deer and/or lower ΔCt values between sika deer and the cross-reacting species.

### Robustness

The robustness of the sika deer-specific real-time PCR assay was tested with DNA isolates from sika deer, red deer and fallow deer (10 ng/µL) and a DNA isolate from a meat extract mixture (5 ng/µL) containing 1% (w/w) sika deer and 99% (w/w) pig. The annealing temperature was varied by ± 1 °C or the volume of the reaction mix (consisting of QuantiTect Multiplex PCR NoROX Master Mix (Qiagen), primer/probe mix and water) per PCR reaction by ± 5%. Measurements were carried out in duplicate. The Ct values obtained for sika deer and the ΔCt values between sika deer and the cross-reacting species were similar to those obtained under the standard conditions, demonstrating the robustness of the real-time PCR assay developed.

### Working range, linear range and amplification efficiency

To determine the working range of the assay, we analysed a sika deer DNA isolate (581 ng/µL) serially diluted in water (1:2 to 1:1,048,576). The standard curve, obtained by plotting the Ct value against the logarithm of the sika deer DNA concentration, was linear over a concentration range from 72.6 µg/mL to 8.9 ng/mL (R^2^ = 0.997). The amplification efficiency, calculated from the slope (−3.57) of the standard curve, was 91%. To evaluate the range of linearity, we analysed a sika deer DNA isolate (20 ng/µL) serially diluted in non-target DNA (pig DNA extract, 20 ng/µL, 1:2 to 1:32,768). Linear relationship (R^2^ = 0.999) was observed between 20 µg/mL and 2.4 ng/mL sika deer DNA. The slope of the standard curve (– 3.42) corresponded to an amplification efficiency of 96%.

### Limit of detection (LOD), limit of quantification (LOQ) and repeatability

**Figure 3:**
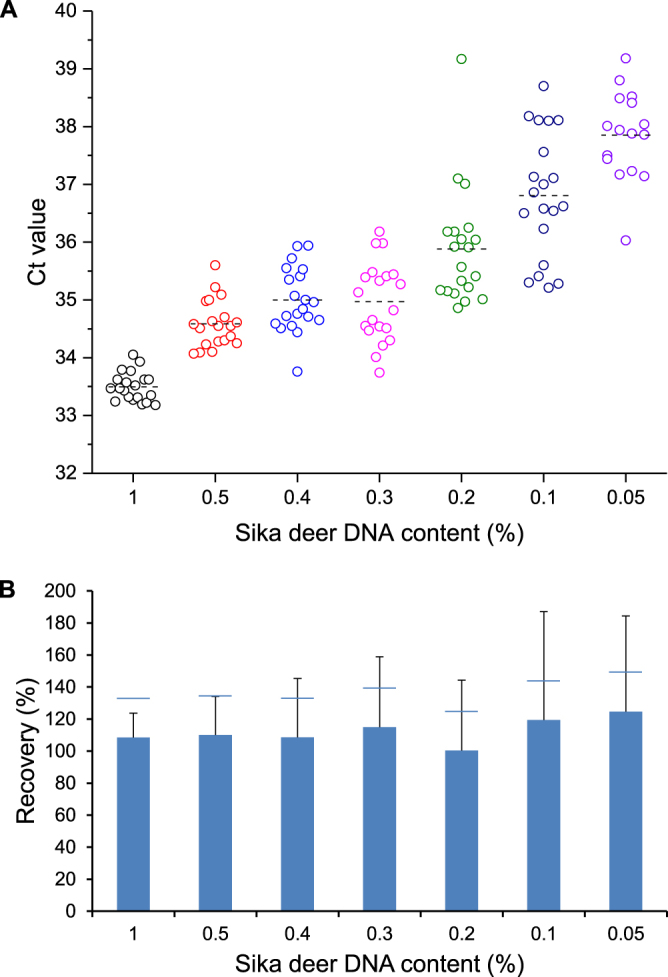
Determination of LOD and LOQ by analysing serial dilutions of a DNA mixture containing 1% (w/w) sika deer and 99% (w/w) pig DNA (non-target DNA). Measurements were carried out in 20 replicates. (**A**) Dot density plot. Circles represent individual Ct values. The horizontal lines indicate the mean values. (**B**) Column plot showing mean recovery and relative standard deviation (RSD). Horizontal lines indicate a RSD of 25%.

LOD and LOQ of the sika deer-specific real-time PCR assay were determined by analysing DNA mixtures (5 ng/µL) containing 1%, 0.5%, 0.4%, 0.3%, 0.2%, 0.1% or 0.05% (w/w) sika deer in pig DNA. The LOD was defined as the lowest sika deer DNA concentration that led to an increase in the fluorescence signal within 36.5 cycles in at least 19 of 20 measurements. With the limit of 36.5 cycles, the Ct value was below the Ct values obtained for DNA isolates (5 ng/µl) from cross-reacting species, in particular red deer (Ct = 36.98), fallow deer (Ct = 38.57), reindeer (Ct = 38.15) and moose (Ct = 39.76). The LOD of the real-time PCR assay was determined to be 0.3% (Fig. 3A). The LOQ, defined as the lowest sika deer DNA concentration that could be determined with a relative standard deviation (RSD) of ≤25%, was found to be 0.5% (RSD 24.1%, Fig. 3B).

Since the genome size of sika deer was not available, we used droplet digital PCR (ddPCR) to relate the DNA amount to the copy number. The droplet number, serving as quality control, was between 12,497 and 17,520 and thus in the range (10,000–20,000) recommended by the supplier of the instrument^22^. The mass of one copy of sika deer genome was determined to be 7.4 pg. Thus, the LOD (0.3%) and the LOQ (0.5%) of the real-time PCR assay corresponded to 10 ± 1 (mean ± SD; n = 13) and 17 ± 2 (mean ± SD; n = 13) copies, respectively. LOD and LOQ had been determined in the presence of approximately 7,300 copies of pig DNA.

The repeatability of the real-time PCR assay was investigated by analysing a serially diluted DNA mixture consisting of 25% (w/w) sika deer and 75% (w/w) pig DNA. Analyses were carried out in a total of five replicates on two different days. The RSD of the Ct values was ≤3.3%, demonstrating the high repeatability of the assay.

### Accuracy

**Figure 4:**
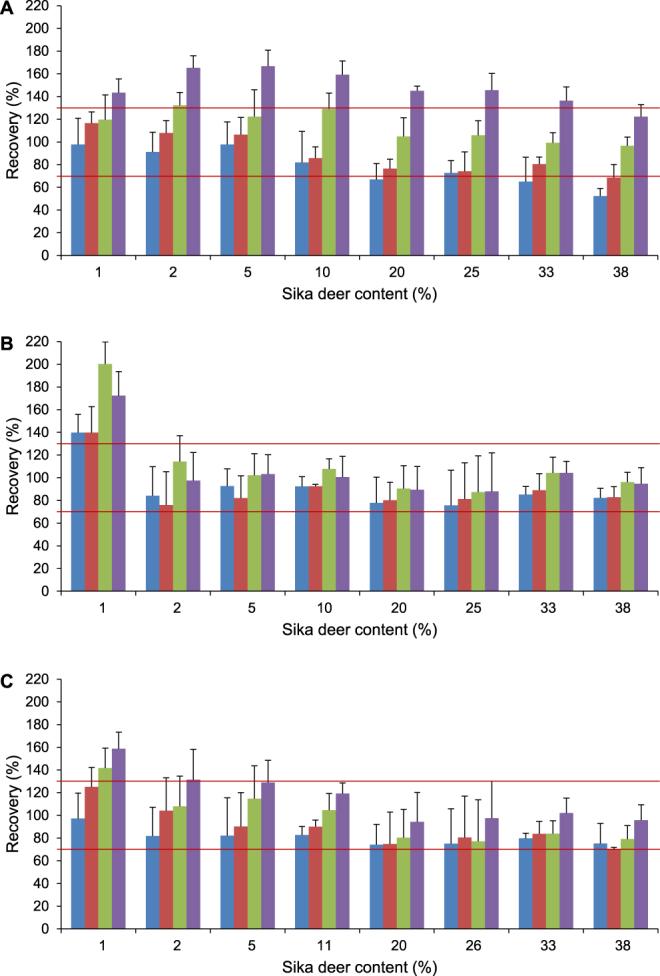
Mean recovery and RSD obtained for (**A**) DNA mixtures, (**B**) meat extract mixtures and (**C**) meat mixtures. Calibration mixtures contained 5% (blue columns), 10% (red columns), 25% (green columns) and 50% (violet columns) sika deer. Red horizontal lines indicate recoveries between 70% and 130%.

To investigate the accuracy of quantitative results, we prepared 33 DNA mixtures, 33 meat extract mixtures and 33 meat mixtures containing sika deer and pig. The sika deer content (DNA, meat extract or meat, respectively) was in the range from 1 to 50% (Supplementary Table 3). To investigate if the presence of a cross-reacting species had an influence on the accuracy, we prepared mixtures that contained red deer and/or fallow deer (DNA, meat extract, meat) in addition to sika deer and pig. By applying a relative quantification approach, we referred the sika deer DNA concentration to the concentration of total meat DNA in order to determine the sika deer content. The total meat DNA concentration was determined with a reference real-time PCR assay published previously^21^. Both the sika deer-specific and the reference real-time PCR assay had to be calibrated. To investigate the influence of the concentration of the calibration mixture on the recovery, we used four mixtures differing in the sika deer content and serial dilutions thereof as calibrators. Figure 4A–C show the recoveries obtained for DNA mixtures, DNA isolates from meat extract mixtures and meat mixtures, respectively. Individual recoveries are given in Supplementary Tables 3–5.

In case of DNA mixtures, calibration mixtures consisting of 50% (w/w) sika deer and 50% (w/w) pig led to recoveries >100%. Calibrators containing 5% (w/w) sika deer and 95% (w/w) pig were found to be applicable for mixtures containing low sika deer concentrations but yielded too low recoveries for mixtures containing sika deer concentrations ≥20%. In general, recoveries obtained with calibration mixtures consisting of 10 or 25% sika deer were in accordance with the ENGL guidelines^23^. Recoveries obtained with calibration mixtures containing 25% sika deer content were more accurate than those obtained with calibration mixtures containing 10% sika deer content. For meat extract mixtures containing 2–38% sika deer, recoveries were in the range from 70 to 130%, being in accordance with the ENGL guidelines. In contrast to DNA mixtures, the sika deer concentration of the calibrator did not have a drastic influence on the recovery. For meat extract mixtures containing only 1% sika deer, recoveries ranged from 140 to 200%. Recoveries obtained for meat mixtures were also in accordance with the ENGL guidelines. As for meat extract mixtures, the sika deer concentration in the calibrators did not have a strong impact on the accuracy of the results (Supplementary Tables 3–5). The recoveries indicated that neither the presence of red deer nor the presence of fallow deer had an impact on the accuracy.

### Applicability to processed meat products

The applicability of the sika deer-specific real-time PCR assay was investigated by analysing commercial meat products declared to contain sika deer meat. We analysed three differently processed foods including a sausage, a smoked sausage and a pâté. The sika deer content was found to be 17.9 ± 0.9% (mean ± SD; n = 8) in the deer sausage, 16.2 ± 0.3% (mean ± SD; n = 8) in the smoked deer sausage and 20.1 ± 2.3% (mean ± SD; n = 8) in the deer pâté. The sika deer-specific real-time PCR assay was found to be applicable to processed food.

## Discussion

In the present study, we aimed to develop a real-time PCR assay for sika deer that can be used for authenticity testing of meat and meat products. To be applicable in practice, the assay should be specific for sika deer without showing cross-reactivity with other species potentially contained in commercial meat products. In addition, the assay should allow the accurate quantification of the sika deer content.

The specificity of a real-time PCR assay critically depends on the target sequence and the primer/probe system. Thus, the selection of the target sequence and the design of the primer/probe system were key steps in the development of the assay. However, we were faced with several difficulties. Most PCR assays for deer authentication presented previously targeted mitochondrial genes. Since the copy number of the mitochondrial genome varies between tissue types and even between cell types, mitochondrial genes are less suitable for accurate quantification than genomic DNA. Thus, the primer/probe system should target a fragment of a single copy gene. To avoid cross-reactivity, we looked for gene sequences that were available not only for sika deer but also for closely related species. The number of sequences in the NCBI database fulfilling these criteria was, however, limited. Finally, we selected the *kappa-casein precursor* gene, the *nanog* pseudogene and the *protein kinase C iota* gene. Flexibility in primer design was also restricted with regard to the length of the target sequence. The target sequence should be as short as possible to allow its detection not only in raw but also in processed foodstuffs. By alignment of the sequences in the CLC Genomics Workbench (Qiagen) we found out that in each of the potential target sequences there was only one sika deer-specific base. For each of the target sequences, we designed one primer/probe system containing the sika deer-specific base at the 3′ penultimate site of the forward primer (primer/probe systems 1a, 2a and 3a, respectively). As suspected, none of the primer/probe systems allowed the discrimination between sika deer and closely related species. To enhance the specificity for sika deer, we pursued a strategy which we had already applied in the development of specific real-time PCR assays for fallow deer^15^ and red deer^16^. The strategy, first described by Cha *et al*. in 1992^24^, is based on modifying the primer sequence by inserting a base mismatch at the position adjacent to the specific base.

In the unmodified forward primer targeting a fragment of the *kappa-casein precursor* gene, primer 1 fw 1, A at the 3′ penultimate site (G**A**A-3′) was the sika deer-specific base. By replacing G at the adjacent position, primers 1 fw 2 (C**A**A-3′), 1 fw 3 (T**A**A-3′) and 1 fw 4 (A**A**A-3′) were obtained. Consequently, the primer variants had a single mismatch with the template from sika deer, but a double mismatch with templates from closely related species. When we subjected the respective primer/probe systems 1b, 1c and 1d to cross-reactivity tests, they turned out to be more specific for sika deer than primer/probe system 1a. The best results were obtained with primer/probe system 1b. In addition to sika deer (Ct 24.75), PCR products were only obtained for red deer (Ct 36.54), fallow deer (Ct 37.71), reindeer (Ct 38.87) and moose (Ct 39.08). Since the ΔCt value between sika deer and the cross-reacting species was ≥11.79, in our opinion cross-reactivity will not limit the applicability for meat authenticity testing in practice. Primer/probe systems 1c and 1d were less specific than primer/probe system 1b (ΔCt between sika deer and the cross-reacting species ≥7.49). By applying the same strategy to the forward primer targeting a fragment of the *nanog* pseudogene, primer 2 fw 1 (A**A**C-3′), we obtained the variants 2 fw 2 (T**A**C-3′), 2 fw 3 (C**A**C-3′) and 2 fw 4 (G**A**C-3′). However, the respective primer/probe systems 2b-2d did not show enhanced specificity for sika deer compared to primer/probe system 2a. With primer/probe system 3a (forward primer 3 fw 1, T**G**T-3′), amplifying a fragment of the *protein kinase C iota* gene, similar Ct values were obtained for sika deer (Ct 27.74) and the species tested for cross-reactivity (Ct ± SD = 28.40 ± 1.90). Primer/probe systems 3b-3d, including the forward primer variants 3 fw 2 (A**G**T-3′), 3 fw 3 (C**G**T-3′) and 3 fw 4 (G**G**T-3′), respectively, turned out to be not applicable for the authentication of sika deer. All primer/probe systems resulted in high Ct values for sika deer (≥34.30) without showing enhanced specificity. Among the 12 primer/probe systems tested, primer/probe system 1b turned out to be most suitable. When we subjected it to further cross-reactivity tests with DNA isolates from 16 animal species and 49 plant species, we did not observe an increase in the fluorescence signal within 40 cycles.

All results discussed above were obtained with the master mix from one supplier. Next, we tested whether the composition of the master mix had an influence on the specificity of primer/probe system 1b. When we analysed the DNA isolate from the 24 animal species with a total of five master mixes by applying the temperature program proposed by the respective supplier, Ct values for sika deer ranged from 24.72 to 26.74. ΔCt values between sika deer and cross-reacting species were in the range from ≥9.09 to ≥13.00. This demonstrates that the master mix has an influence on the specificity of the real-time PCR assay and thus it has to be selected carefully. However, the thermal cycling program (fast or conventional) did not have an influence, neither on the Ct values obtained for sika deer nor on the ΔCt values between sika deer and cross-reacting species. In addition, the primer and probe concentration is known to affect the amplification efficiency. Experiments performed with a forward primer, reverse primer and probe concentration ratio of 200 nM: 200 nM: 100 nM led to the lowest Ct value for sika deer and the highest ΔCt values between sika deer and the cross-reacting species. Another important influence is the annealing temperature. In the present study, the annealing temperature was kept constant at 60 °C, because it is used in routine real-time PCR analysis at the Austrian Agency for Health and Food Safety (AGES, Vienna, Austria). To test the robustness of the sika deer real-time PCR assay, we analysed DNA isolates from sika deer, red deer and fallow deer under conditions slightly deviating from the standard conditions. Neither variation of the annealing temperature by ± 1 °C nor variation of the volume of the reaction mix per PCR reaction by ± 5% had an influence on the Ct values for the three deer species, demonstrating the robustness of the real-time PCR assay developed.

We characterized and validated the assay by determining main analytical parameters including working range, linear range, LOD, LOQ, repeatability and accuracy. The working range, determined by analysing the sika deer DNA isolate serially diluted in water, was between 72.6 µg/mL and 8.9 ng/mL (R^2^ = 0.997). A similar working range (42.1 µg/mL–10.3 ng/mL) had been obtained for the fallow deer-specific real-time PCR published previously^15^. The range of linearity, evaluated by analysing serial dilutions of the sika deer DNA isolate with pig DNA, was between 20 µg/mL and 2.4 ng/mL. The range of linearity was similar to that of the fallow deer-specific real-time PCR assay (20 µg/mL–9.8 ng/mL). LOD and LOQ were determined by repeatedly (n = 20) analysing DNA mixtures of sika deer and pig DNA. With 0.3% and 0.5%, respectively, LOD and LOQ were slightly higher than LOD (0.1%) and LOQ (0.4%) of the fallow deer-specific assay. We calculated the copy number present at the LOD and LOQ of the sika deer-specific real-time PCR assay using ddPCR. At the LOD, approximately 10 ± 1 (mean ± SD, n = 13) copies and at the LOQ, 17 ± 2 (mean ± SD, n = 13) copies were detectable. This findings are in agreement with long-term experienced practical values for LOD (between 5–10 copies) and LOQ (between 40–100 copies)^25^. In addition, the LOD of the sika deer-specific real-time PCR assay meets the requirements defined by the European Network of GMO Laboratories (ENGL, <25 copies)^23^. RSD of Ct values obtained by analysing DNA mixtures (25% (w/w) sika deer and 75% (w/w) pig DNA) in a total of five replicates on two different days was ≤3.3%. The repeatability was comparable to that of the fallow deer-specific assay (RSD ≤ 2%).

After calibration, the real-time PCR assay could be used to determine the sika deer DNA concentration. However, the sika deer DNA concentration does not necessarily correlate with the sika deer meat content, expressed as weight per weight (w/w)^20^. To avoid systematic errors, we applied a relative quantification strategy. In addition to the sika deer-specific real-time PCR assay, the DNA isolates were subjected to a reference real-time PCR assay. The reference assay, allowing the determination of the total meat DNA by targeting a 70 bp fragment of the myostatin gene, was published recently^21^. The accuracy of our quantification strategy was investigated by analysing 33 DNA mixtures, 33 meat extract mixtures and 33 meat mixtures. All mixtures contained sika deer (1% – 50%) and pig, part of them additionally red deer and/or fallow deer. With the exception of mixtures consisting of only 1% sika deer, recoveries were in the range from 70 to 130%. Thus, the accuracy of the assay meets the demands of the guidelines established by ENGL. Neither the presence of red deer nor the presence of fallow deer had an impact on the accuracy. However, in case of DNA mixtures, calibration mixtures consisting of 50% (w/w) sika deer and 50% (w/w) pig led to recoveries ≥130%. These results indicate that the sika deer concentration in the calibrator should be adapted to the sika deer concentration in the samples. Therefore, we recommend starting with a preliminary analysis of the sample together with a cut-off standard. If the sika deer concentration in the cut-off standard is near the LOD, it serves as positive control and allows the rough estimation of the sika deer content in the sample. Based on this information, adequate calibration mixtures can be prepared and used for the subsequent, more accurate quantitative analysis.

Furthermore, to test whether our sika deer-specific real-time PCR assay was applicable to processed meat products, we analysed commercial game meat products containing sika deer according to the manufacturer. The products, a sausage, a smoked sausage and a pâté, differed in the processing type. The real-time PCR assay enabled the detection and quantification of sika deer in each of the three products.

## Materials and Methods

### Samples

Game meat samples were collected from the Research Institute of Wildlife Ecology (Vienna, Austria), the Wildpark Ernstbrunn (Ernstbrunn, Austria) and the University of Veterinary Medicine Vienna (Vienna, Austria). Meat from domestic animals was obtained from local meat markets and supermarkets. All samples originated from fresh and lean muscle meat. Meat samples were stored at −20 °C for further use. The identity of the animal species was confirmed by sequencing. “Meat mixtures” were produced by weighing out and mixing meat of the respective animal species before extracting and isolating the DNA. “Meat extract mixtures” were prepared by mixing the extracts from meat flesh of the respective animal species before DNA isolation. “DNA mixtures” were prepared by mixing DNA isolates from muscle meat of the respective animal species.

In addition, three commercially available processed game meat products were analysed, a deer sausage, a smoked deer sausage and a deer pâté. According to the manufacturer these products contained sika deer meat. The deer sausage was declared to contain 67% deer meat, 33% pig meat and spices. According to the ingredient list, the smoked deer sausage consisted of 55% deer meat, 18% bacon, 16% beef meat, 11% pig meat, spices, iodised table salt, dextrose and preservative sodium nitrite (pasteurised). The deer pâté was declared to contain 51% deer meat, 48% pig meat, spices, onion, garlic and table salt (pasteurised).

### DNA isolation

DNA isolation was performed with a CTAB protocol as described previously^13^. Concentration and purity of the isolated DNA were determined from the absorbance values at 260 nm and 280 nm (QIAxpert spectrophotometer, Qiagen). DNA isolates were stored at -20 °C.

### Primers and probes

Genomic DNA sequences were downloaded from the NCBI database^26^. Sequence similarity was detected via online BLAST (Basic Local Alignment Algorithm Search Tool)^27^, sequence alignment was performed with the CLC Genomics Workbench 8.0 (Qiagen). Primers and probes were designed with Primer Express 3.0 (Applied Biosystems). Probes were labelled with DFO (Dragonfly Orange) at the 5′ end and a non-fluorescent minor groove binding quencher (MGBNFQ) at the 3′ end. Primers were synthesised by Sigma Aldrich (Darmstadt, Germany), probes by Eurogentec. All primer and probe sequences tested are given in Supplementary Table 1.

### Real-time PCR

Real-time PCR was performed in an optical 96-well reaction plate (0.2 mL, Applied Biosystems) sealed with an optical adhesive film (Applied Biosystems) on the ABI 7500 Real-time PCR System (Applied Biosystems). The total reaction volume was kept constant at 25 µL. Unless indicated differently, the reaction mix consisted of 12.5 µL QuantiTect Multiplex PCR NoROX Master Mix (Qiagen), 2.5 µL ultrapure water, 5 µL 5-fold concentrated primer/probe mix and 5 µL DNA isolate (DNA concentration between 5 ng/µL and 20 ng/µL). In order to use the QuantiTect Multiplex PCR NoROX Master Mix on the ABI 7500 Real-time PCR System, 2 µL ROX Reference Dye (25 µM) (Invitrogen by Life Technologies, Carlsbad, CA, USA) were added to 1.8 mL master mix. As a result, the final ROX concentration was 14 nM per PCR reaction. The standard temperature program started with a denaturation step of 15 min at 95 °C, followed by 40 cycles of 1 min at 94 °C and 1 min at 60 °C. This temperature program was used if not indicated otherwise.

### Specificity of the primer/probe systems

All primer/probe systems were tested for cross-reactivity with DNA isolates (10 ng/µL) from the following 23 animal species: alpine ibex (*Capra ibex*), cattle (*Bos taurus*), chamois (*Rupicapra rupicapra*), chicken (*Gallus gallus*), crocodile (*Crocodylus niloticus*), duck (*Anatidae*), fallow deer (*Dama dama*), goat (*Capra hircus*), goose (*Anserinae*), hare (*Lepus europaeus*), horse (*Equus caballus*), kangaroo (*Macropodidae*), moose (*Alces alces*), mouflon (*Ovis orientalis*), ostrich (*Struthio camelus*), pig (*Sus scrofa domestica*), rabbit (*Oryctolagus cuniculus*), red deer (*Cervus elaphus*), reindeer (*Rangifer tarandus*), roe deer (*Capreolus capreolus*), sheep (*Ovis aries*), turkey (*Meleagris gallopavo*) and wild boar (*Sus scrofa scrofa*).

In further experiments, primer/probe system 1b (Supplementary Table 1) was tested for cross-reactivity with DNA isolates (20 ng/µL) from the following 49 plant species: allspice (*Pimenta dioica*), anise (*Pimpinella anisum*), bay leaf (*Laurus nobilis*), bean (*Phaseolus vulgaris*), black mustard (*Brassica nigra*), black pepper (*Piper nigrum*), broccoli (*Brassica oleracea*), buckwheat (*Fagopyrum esculentum*), caraway (*Carum carvi*), cardamom (*Elettaria cardamomum*), carrot (*Daucus carota*), celery (*Apium graveolens*), chili pepper (*Capsicum sp*.), chives (*Allium schoenoprasum*), coriander (*Coriandrum sativum*), cumin (*Cuminum cyminum*), curcuma (*Curcuma longa/domestica*), dill (*Anethum graveolens*), fennel (*Foeniculum vulgare*), garlic (*Allium sativum*), green pea (*Pisum sativum*), horseradish (*Armoracia rusticana*), leek (*Allium ampeloprasum*), lentil (*Lens culinaris*), lovage (*Levisticum officinale*), maize (*Zea mays*), marjoram (*Origanum majorana*), onion (*Allium cepa*), oregano (*Origanum vulgare*), parsley (*Petroselinum crispum*), parsnip (*Pastinaca sativa*), peanut (*Arachis hypogaea*), pearl millet (*Pennisetum glaucum*), potato (*Solanum tuberosum*), radish (*Raphanus sativus*), rapeseed (*Brassica napus*), rice (*Oryza sativa*), rosemary (*Rosmarinus officinalis*), rye (*Secale cereale*), sage (*Salvia officinalis*), savory (*Satureja hortensis*), sesame (*Sesamum indicum*), sweet pepper (*Capsicum annuum*), tarragon (*Artemisia dracunculus*), thyme (*Thymus vulgaris*), tomato (*Solanum lycopersicum*), walnut (*Juglans regia*), wheat (*Triticum aestivum*) and white mustard (*Sinapis alba*).

Cross-reactivity tests were carried out with the QuantiTect® Multiplex PCR NoROX Master Mix (Qiagen). The thermal conditions were: initial denaturation 15 min 95 °C, followed by 40 × (1 min 94 °C, 1 min 60 °C). The concentration of forward primer, reverse primer and probe was 200 nM, 200 nM and 100 nM, respectively.

Primer/probe system 1b was subjected to further cross-reactivity tests by analysing the DNA isolates from the 23 animal species with other commercial master mixes. The following master mixes were tested (the respective temperature protocols are given in bracket): TaqMan® Universal PCR Master Mix (Applied Biosystems; 10 min 95 °C, 45× (15 s 95 °C, 1 min 60 °C)); GoTaq® Probe qPCR Master Mix (Promega; 2 min 95 °C, 40× (3 s 95 °C, 31 s 60 °C)); PerfeCTa® qPCR ToughMix^TM^, Low ROX^TM^ (Quanta Biosciences; 10 min 95 °C, 45 × (5 s 95 °C, 31 s 60 °C)) and Takyon^TM^ No Rox Probe MasterMix dTTP Blue (Eurogentec; 3 min 95 °C, 40× (3 s 95 °C, 31 s 60 °C)). To master mixes that did not a priori contain a passive reference dye, a passive reference dye was added, either following the manufacturer’s suggestions or according to our practical experience. We added 2 µL CXR dye (30 µM, included in the master mix kit) to 1 mL of the GoTaq® Probe qPCR Master Mix (Promega) and 2 µL ROX Reference Dye (25 µM) (Invitrogen by Life Technologies) to 1 mL of the Takyon^TM^ No Rox Probe MasterMix dTTP Blue (Eurogentec), resulting in final CXR and ROX reference dye concentrations of 30 nM and 25 nM per PCR reaction, respectively.

### Optimisation of the concentration of primer/probe system 1b

DNA isolates (10 ng/µL) from sika deer, red deer and fallow deer were used to optimise the concentration of primer/probe system 1b. The following combinations (forward primer/ reverse primer/ probe concentration in nM) were tested: 100/100/50, 100/100/100, 100/100/250, 200/200/50, 200/200/100, 200/200/250, 200/100/100, 350/350/50, 350/350/100, 350/350/250, 400/100/100, 400/200/100, 500/500/50, 500/500/100, 500/500/250, 600/100/100, 600/200/100, 700/700/50, 700/700/100, 700/700/250, 800/100/100, 800/200/100, 900/900/50, 900/900/100 and 900/900/250.

### Robustness of the real-time PCR assay

For testing the robustness of the real-time PCR assay, the annealing temperature was varied by ±1 °C and the volume of the reaction mix (containing QuantiTect® Multiplex PCR NoROX Master Mix, ultrapure water, primers and probe) by ± 5%. These experiments were carried out with DNA isolates from sika deer, red deer and fallow deer (10 ng/µL) and, to be near the LOQ^23^, with a DNA isolate of a meat extract mixture (5 ng/µL) containing 1% (w/w) sika deer and 99% (w/w) pig. All measurements were performed in duplicate.

### Working range, linear range and amplification efficiency

The working range of the real-time PCR assay was determined by analysing a sika deer DNA isolate (581 ng/µL) serially diluted in water (1:2 to 1:1,048,576). The linear range was determined by analysing a sika deer DNA isolate (20 ng/µL) serially diluted (1:2 to 1:32,768) with pig DNA (20 ng/µL).

After plotting the Ct values against the logarithm of the sika deer DNA concentrations, the equation of the standard curve was obtained by linear regression. From the slope of the standard curve, the amplification efficiency was calculated as follows:

1 $$E( \% )=({10}^{(\frac{-1}{slope})}-1)\cdot 100.$$

### LOD, LOQ and repeatability

LOD and LOQ of the real-time PCR assay were determined following the recommendations of the European Network of GMO Laboratories^23^. A DNA isolate (5 ng/µL), containing 1% (w/w) sika deer and 99% (w/w) non-target pig DNA, was serially diluted with pig DNA (5 ng/µL) in order to obtain mixtures containing 0.5%, 0.4%, 0.3%, 0.2%, 0.1% and 0.05% (w/w) sika deer DNA. The LOD was defined as the lowest concentration that led to an increase in the fluorescence signal in at least 19 out of 20 experiments. In addition, the Ct value obtained had to be below that obtained for DNA isolates (5 ng/µL) from cross-reacting species.

After calibrating the real-time PCR assay by analysing a DNA mixture (20 ng/µL) containing 25% (w/w) sika deer DNA in pork DNA and serial dilutions (1:4 to 1:1,024) thereof in water in triplicates, the LOQ was determined as the lowest concentration for which a RSD of ≤25% was achieved.

The repeatability of the assay was investigated by analysing a DNA mixture containing 25% (w/w) sika deer in pig DNA (20 ng/µL) and five dilutions thereof (1:4 to 1:1,024) in five replicates on two days.

### Assessment of the copy number at the LOD and LOQ by ddPCR

ddPCR was used to assess the number of target copies present at the LOD and LOQ of our real-time PCR assay. Six sika deer DNA isolates (5 ng/µL) were analysed in a total of 13 replicates. Primer/probe system 1b was used as given in Supplementary Table 1, with the exception that the probe was labelled with FAM instead of DFO. The primer and probe concentrations were the same as applied for real-time PCR (200 nM forward primer, 200 nM reverse primer, 100 nM probe). The reaction mix consisted of 12.5 µL ddPCR Supermix for Probes (No dUTP) (Bio-Rad, Hercules, CA, USA), 1.25 µL 20-fold primer/probe mix, 1.25 µL ultrapure water and 10 µL DNA extract (5 ng/µL) or ultrapure water for the no-template control (NTC). A 20 µL aliquot of this 25 µL reaction mix was subsequently subjected to droplet generation in a QX200 Droplet Generator (Bio-Rad), using 70 µL Droplet Generation Oil for Probes (Bio-Rad), DG8 Cartridges for QX100/QX200 Droplet Generator (Bio-Rad) and Droplet Generator DG8 Gasket (Bio-Rad). After droplet generation, the entire product was transferred to a twin.tec Plate 96, semi-skirted, colourless (Eppendorf, Hamburg, Germany) and sealed with a Pierceable Foil Heat Seal (Bio-Rad) in a PX1 PCR Plate Sealer (Bio-Rad). PCR was performed in a Mastercycler ep gradient S (Eppendorf) applying the following temperature program: denaturation step at 95 °C for 10 min, followed by 40 cycles at 94 °C for 30 sec and 60 °C for 1 min, and a final enzyme deactivation step at 98 °C for 10 min. All ramp rates were set to 50%. Subsequently, the droplets were read out on a QX200 Droplet Reader (Bio-Rad). Data analysis was performed using QuantaSoft 1.7.4 (Bio-Rad).

### Quantification

The sika deer content was determined relatively by analysing the DNA isolates by the sika deer-specific real-time PCR assay and a reference real-time PCR assay allowing the determination of the total amount of mammalian and poultry species^21^. For the application to DNA mixtures, both real-time PCR assays were calibrated with DNA mixtures containing 50%, 25%, 10% or 5% (w/w) sika deer DNA in pig. For the determination of the sika deer content in meat extract mixtures, DNA isolates from meat extract mixtures containing 50%, 25%, 10% or 5% (w/w) sika deer in pig were used for calibration. For the analysis of meat mixtures, the real-time PCR assays were calibrated with DNA isolates from meat mixtures containing 50%, 28%, 10% or 5% (w/w) sika deer in pig. To quantify the sika deer content in the commercial game meat products, both assays were calibrated with a meat extract mixture containing 50% (w/w) sika deer and 50% (w/w) pig. All calibration mixtures were adjusted to a DNA concentration of 20 ng/µL, serially diluted (1:4; 1:16; 1:64; 1:256 and 1:1,024) in water. DNA isolates from samples were diluted to 5 ng/µL.

The percentage of sika deer was calculated using the following equations:

2 $${c}_{DNAsikadeer}(\frac{{\rm{ng}}}{\mu {\rm{L}}})={10}^{\frac{C{t}_{spec.}-{d}_{spec.}}{slop{e}_{spec.}}}$$

3 $${c}_{DNAtotalmeat}(\frac{{\rm{ng}}}{\mu {\rm{L}}})={10}^{\frac{C{t}_{ref.}-{d}_{ref.}}{slop{e}_{ref.}}}$$

4 $$conten{t}_{sikadeer}\,( \% )=\frac{{c}_{DNAsikadeer}(\frac{{\rm{ng}}}{\mu \,{\rm{L}}})}{{c}_{DNAtotalmeat}(\frac{{\rm{ng}}}{\mu \,{\rm{L}}})}\cdot 100$$

where c_DNA sika deer_, c_DNA total meat_ are the concentrations of the sika deer DNA and total meat DNA, respectively; Ct_spec._, Ct_ref._ are the Ct values received by using the sika deer-specific and the reference real-time PCR assay, respectively; d_spec._, d_ref._ are the intercepts of the standard curves of the sika deer-specific and the reference real-time PCR assay, respectively, and slope_spec._, slope_ref._ are the slopes of the calibration curves of the sika deer-specific and the reference real-time PCR assay, respectively.

The recovery was calculated by referring the sika deer content determined by real-time PCR to the theoretical sika deer content using the following equation:

5 $$Recovery\,R\,( \% )=\frac{determined\,sika\,deer\,content\,( \% )}{theoretical\,sika\,deer\,content\,( \% )}\cdot 100$$

### Influence of the presence of red deer and fallow deer on the accuracy of the real-time PCR assay

To investigate if the accuracy of the quantification results is influenced by the presence of red deer or fallow deer, DNA mixtures, meat extract mixtures and meat mixtures containing different amounts (in the range from 0% to 39%) of red deer and fallow deer (DNA/ meat extract/ meat) were produced. DNA isolates obtained from these mixtures were diluted to a DNA concentration of 5 ng/µL and analysed by the sika deer-specific and the reference real-time PCR assay.

## Electronic supplementary material

Supplementary material
