# Supplementary material for: Sika deer (Cervus nippon)-specific real-time PCR method to detect fraudulent labelling of meat and meat products
Source: Sci Rep. 2018 May 8;8:7236. doi: 10.1038/s41598-018-25299-7 (PMC5940659; doi:10.1038/s41598-018-25299-7)
Supplement: Supplementary file 1 — Supplementary material [file 41598_2018_25299_MOESM1_ESM.docx]

**Sika deer (*Cervus nippon*)-specific real-time PCR method to detect fraudulent labelling of meat and meat products**

Maria Kaltenbrunner^1,2^, Rupert Hochegger^1^ and Margit Cichna-Markl^2,*^

^1^ Austrian Agency for Health and Food Safety, Institute for Food Safety Vienna, Department of Molecular Biology and Microbiology, Spargelfeldstraße 191, 1220 Vienna, Austria

^2^ Department of Analytical Chemistry, Faculty of Chemistry, University of Vienna, Währinger Straße 38, 1090 Vienna, Austria

***Corresponding author**

Tel: +43-1-4277-52374

Fax: +43-1-4277-9523

E-mail: [margit.cichna@univie.ac.at](mailto:margit.cichna@univie.ac.at)

**The co-authors e-mail addresses:**

Maria Kaltenbrunner: [maria.kaltenbrunner@ages.at](mailto:maria.kaltenbrunner@ages.at)

Rupert Hochegger: [rupert.hochegger@ages.at](mailto:rupert.hochegger@ages.at)

Supplementary Table 1: Primer and probe sequences tested in this study.

| **Gene (NCBI accession number)** | **System** | **Primer/probe sequence (5' - 3')** | | **Length [nt]** | **Amplicon [nt]** |
| --- | --- | --- | --- | --- | --- |
| *Gene for kappa-casein precursor,* | 1a | 1 fw 1 | GGTGCACTCTCAATAACTTCTGAG**A**A | 26 | 71 |
| *complete cds* (D14379.2) |  | 1 rev | CCTACCACCGAAGCAATAGTGG | 22 |  |
|  |  | 1 probe | GCTTCTTGAGTAGCTACAGTG | 21 |  |
|  | 1b | 1 fw 2 | GGTGCACTCTCAATAACTTCTGAC**A**A | 26 | 71 |
|  |  | 1 rev | CCTACCACCGAAGCAATAGTGG | 22 |  |
|  |  | 1 probe | GCTTCTTGAGTAGCTACAGTG | 21 |  |
|  | 1c | 1 fw 3 | GGTGCACTCTCAATAACTTCTGAT**A**A | 26 | 71 |
|  |  | 1 rev | CCTACCACCGAAGCAATAGTGG | 22 |  |
|  |  | 1 probe | GCTTCTTGAGTAGCTACAGTG | 21 |  |
|  | 1d | 1 fw 4 | GGTGCACTCTCAATAACTTCTGAA**A**A | 26 | 71 |
|  |  | 1 rev | CCTACCACCGAAGCAATAGTGG | 22 |  |
|  |  | 1 probe | GCTTCTTGAGTAGCTACAGTG | 21 |  |
| *Nanog pseudogene,* | 2a | 2 fw 1 | GGGGTTATTCCAGGTCTGGTTA**A**C | 24 | 65 |
| *complete sequence* |  | 2 rev | GGGATGTTTTGGTGAACTCTCCT | 23 |  |
| (KR005602.1) |  | 2 probe | ATGGGCAGGTTTC | 13 |  |
|  | 2b | 2 fw 2 | GGGGTTATTCCAGGTCTGGTTT**A**C | 24 | 65 |
|  |  | 2 rev | GGGATGTTTTGGTGAACTCTCCT | 23 |  |
|  |  | 2 probe | ATGGGCAGGTTTC | 13 |  |
|  | 2c | 2 fw 3 | GGGTTATTCCAGGTCTGGTTC**A**C | 23 | 64 |
|  |  | 2 rev | GGGATGTTTTGGTGAACTCTCCT | 23 |  |
|  |  | 2 probe | ATGGGCAGGTTTC | 13 |  |
|  | 2d | 2 fw 4 | GGGTTATTCCAGGTCTGGTTG**A**C | 23 | 64 |
|  |  | 2 rev | GGGATGTTTTGGTGAACTCTCCT | 23 |  |
|  |  | 2 probe | ATGGGCAGGTTTC | 13 |  |
| *Protein kinase C iota (PRKCI)* | 3a | 3 fw 1 | CTTATATATTAAGCAGTCTGAAGCATGTAT**G**T | 32 | 79 |
| *gene, partial sequence* |  | 3 rev | AGAAAAAATGTCAATGTTTGTGCC | 24 |  |
| (DQ379332.1) |  | 3 probe | AGCAGACAGAAACAAT | 16 |  |
|  | 3b | 3 fw 2 | CTTATATATTAAGCAGTCTGAAGCATGTAA**G**T | 32 | 79 |
|  |  | 3 rev | AGAAAAAATGTCAATGTTTGTGCC | 24 |  |
|  |  | 3 probe | AGCAGACAGAAACAAT | 16 |  |
|  | 3c | 3 fw 3 | CTTATATATTAAGCAGTCTGAAGCATGTAC**G**T | 32 | 79 |
|  |  | 3 rev | AGAAAAAATGTCAATGTTTGTGCC | 24 |  |
|  |  | 3 probe | AGCAGACAGAAACAAT | 16 |  |
|  | 3d | 3 fw 4 | CTTATATATTAAGCAGTCTGAAGCATGTAG**G**T | 32 | 79 |
|  |  | 3 rev | AGAAAAAATGTCAATGTTTGTGCC | 24 |  |
|  |  | 3 probe | AGCAGACAGAAACAAT | 16 |  |
| Bold letters in the primer and probe sequences indicate sika deer-specific bases | | | | | |
| Underlined letters in the primer and probe sequences indicate mismatch bases | | | | | |

Supplementary Table 2: Results of the preliminary specificity tests carried out using 12 different primer/probe systems with DNA isolates from eight animal species (10 ng/µL). Measurements were carried out in n ≥ 2 replicates.

| **Primer/probe system** | | **1a** | **1b** | **1c** | **1d** | **2a** | **2b** | **2c** | **2d** | **3a** | **3b** | **3c** | **3d** |
| --- | --- | --- | --- | --- | --- | --- | --- | --- | --- | --- | --- | --- | --- |
| **Species** | **Binomial name** | **Mean**  **Ct value** | **Mean**  **Ct value** | **Mean**  **Ct value** | **Mean**  **Ct value** | **Mean**  **Ct value** | **Mean**  **Ct value** | **Mean**  **Ct value** | **Mean**  **Ct value** | **Mean**  **Ct value** | **Mean**  **Ct value** | **Mean**  **Ct value** | **Mean**  **Ct value** |
| Sika deer | *Cervus nippon* | 23.50 | 24.75 | 24.49 | 24.30 | 24.17 | 24.09 | 29.03 | 29.15 | 27.74 | 35.32 | 34.30 | 38.81 |
| Red deer | *Cervus elaphus* | 22.97 | 36.54 | 31.97 | 32.53 | 23.98 | 23.93 | 29.03 | 29.01 | 27.40 | 35.37 | 34.17 | 38.69 |
| Fallow deer | *Dama dama* | 24.56 | 37.71 | 33.62 | 34.38 | 23.88 | 23.87 | 29.11 | 29.17 | 28.05 | 35.64 | 34.76 | - ^a^ |
| Roe deer | *Capreolus capreolus* | 31.44 | - ^a^ | - ^a^ | - ^a^ | 23.40 | 24.36 | 29.55 | 29.29 | 27.64 | 35.15 | 34.18 | 37.95 |
| Alpine ibex | *Capra ibex* | -^a^ | - ^a^ | - ^a^ | - ^a^ | 23.52 | 24.22 | 29.16 | 29.11 | - ^a^ | - ^a^ | - ^a^ | - ^a^ |
| Reindeer | *Rangifer tarandus* | 26.63 | 38.87^b^ | 34.70 | 36.11 | 25.57 | 26.35 | 31.07 | 30.80 | 31.74 | 39.46^c^ | 38.38 | - ^a^ |
| Moose | *Alces alces* | 25.78 | 39.08^b^ | 34.40 | 35.09 | 22.45 | 23.12 | 27.42 | 27.22 | 27.14 | 34.77 | 33.50 | 37.60 |
| Goat | *Capra hircus* | -^a^ | - ^a^ | - ^a^ | - ^a^ | 24.15 | 24.81 | 30.16 | 29.99 | - ^a^ | - ^a^ | - ^a^ | - ^a^ |
| ^a^ No increase in the fluorescence signal within 40 cycles. | | | | | | | | | | | | | |
| ^b^ Only three out of four replicates led to an increase in the fluorescence signal within 40 cycles. | | | | | | | | | | | | | |
| ^c^ Only one out of two replicates led to an increase in the fluorescence signal within 40 cycles. | | | | | | | | | | | | | |

Supplementary Table 3: Quantification results obtained for DNA mixtures. DNA mixtures consisting of sika deer (50%, 25%, 10% or 5%) and pig were used for calibration. Analyses were performed in duplicates.

| **Content** | | | |  | **Calibrator 50% sika deer** | |  | **Calibrator 25% sika deer** | |  | **Calibrator 10% sika deer** | |  | **Calibrator 5% sika deer** | |
| --- | --- | --- | --- | --- | --- | --- | --- | --- | --- | --- | --- | --- | --- | --- | --- |
| **Sika deer  (%)** | **Red deer  (%)** | **Fallow deer (%)** | **Pig  (%)** |  | **Mean sika deer content determined (%)** | **Mean recovery (%)** |  | **Mean sika deer content determined (%)** | **Mean recovery (%)** |  | **Mean sika deer content determined (%)** | **Mean recovery (%)** |  | **Mean sika deer content determined (%)** | **Mean recovery (%)** |
| **DNA mixtures** | | | | | | | | | | | | | | | |
| 1 | 0 | 0 | 99 |  | 1.4 | 139 |  | 1.2 | 118 |  | 1.0 | 102 |  | 1.3 | 129 |
| 1 | 1 | 1 | 97 |  | 1.6 | 159 |  | 1.6 | 155 |  | 1.2 | 124 |  | 1.0 | 101 |
| 1 | 19 | 19 | 61 |  | 1.2 | 121 |  | 0.9 | 93 |  | 1.1 | 113 |  | 0.8 | 82 |
| 1 | 0 | 1 | 98 |  | 1.6 | 155 |  | 1.1 | 112 |  | 1.3 | 128 |  | 0.8 | 80 |
| 2 | 0 | 0 | 98 |  | 3.6 | 182 |  | 2.3 | 114 |  | 2.6 | 128 |  | 1.6 | 78 |
| 2 | 2 | 0 | 96 |  | 3.3 | 164 |  | 3.0 | 148 |  | 2.0 | 98 |  | 2.3 | 117 |
| 2 | 2 | 2 | 94 |  | 3.6 | 178 |  | 2.9 | 146 |  | 2.0 | 101 |  | 1.5 | 77 |
| 2 | 38 | 0 | 60 |  | 3.6 | 178 |  | 2.8 | 139 |  | 2.1 | 104 |  | 2.1 | 103 |
| 2 | 0 | 38 | 60 |  | 3.0 | 149 |  | 2.3 | 115 |  | 2.3 | 117 |  | 1.7 | 86 |
| 2 | 19 | 19 | 60 |  | 2.8 | 140 |  | 2.7 | 133 |  | 2.0 | 101 |  | 1.7 | 86 |
| 5 | 33 | 0 | 62 |  | 7.3 | 145 |  | 5.6 | 111 |  | 4.3 | 87 |  | 4.3 | 85 |
| 5 | 15 | 15 | 65 |  | 7.7 | 154 |  | 3.7 | 75 |  | 5.7 | 113 |  | 4.0 | 80 |
| 5 | 0 | 0 | 95 |  | 8.5 | 170 |  | 7.0 | 141 |  | 5.6 | 112 |  | 6.4 | 129 |
| 5 | 5 | 0 | 90 |  | 9.6 | 192 |  | 7.3 | 146 |  | 6.6 | 132 |  | 5.7 | 113 |
| 5 | 5 | 5 | 85 |  | 9.9 | 197 |  | 7.5 | 150 |  | 4.8 | 96 |  | 4.8 | 96 |
| 5 | 0 | 33 | 62 |  | 7.1 | 142 |  | 5.6 | 111 |  | 4.9 | 98 |  | 4.2 | 84 |
| 10 | 0 | 0 | 90 |  | 16.9 | 169 |  | 11.2 | 112 |  | 8.6 | 86 |  | 7.7 | 77 |
| 10 | 10 | 0 | 80 |  | 13.7 | 137 |  | 12.7 | 127 |  | 7.7 | 77 |  | 10.6 | 106 |
| 10 | 10 | 10 | 70 |  | 17.2 | 172 |  | 14.8 | 148 |  | 9.4 | 94 |  | 6.2 | 62 |
| 20 | 0 | 0 | 80 |  | 29.6 | 148 |  | 23.1 | 115 |  | 15.4 | 77 |  | 12.7 | 64 |
| 20 | 20 | 0 | 60 |  | 29.6 | 148 |  | 23.6 | 118 |  | 16.6 | 83 |  | 16.1 | 80 |
| 20 | 0 | 20 | 60 |  | 29.7 | 148 |  | 16.0 | 80 |  | 13.5 | 68 |  | 13.0 | 65 |
| 20 | 20 | 20 | 40 |  | 27.2 | 136 |  | 21.1 | 106 |  | 15.6 | 78 |  | 11.7 | 58 |
| 25 | 0 | 0 | 75 |  | 41.6 | 166 |  | 29.1 | 116 |  | 18.0 | 72 |  | 20.4 | 82 |
| 25 | 25 | 0 | 50 |  | 36.8 | 147 |  | 27.7 | 111 |  | 22.0 | 88 |  | 16.6 | 66 |
| 25 | 25 | 25 | 25 |  | 30.9 | 124 |  | 22.6 | 90 |  | 15.7 | 63 |  | 17.6 | 70 |
| 33 | 0 | 0 | 67 |  | 41.2 | 125 |  | 36.0 | 109 |  | 25.8 | 78 |  | 22.5 | 68 |
| 33 | 5 | 0 | 62 |  | 42.6 | 129 |  | 30.7 | 93 |  | 28.5 | 86 |  | 16.4 | 50 |
| 33 | 5 | 5 | 57 |  | 51.2 | 155 |  | 31.5 | 95 |  | 25.4 | 77 |  | 25.5 | 77 |
| 38 | 0 | 0 | 62 |  | 50.0 | 132 |  | 34.6 | 91 |  | 28.2 | 74 |  | 20.8 | 55 |
| 38 | 5 | 5 | 52 |  | 42.9 | 113 |  | 38.7 | 102 |  | 24.0 | 63 |  | 18.9 | 50 |
| 34 | 33 | 33 | 0 |  | 40.7 | 120 |  | 29.9 | 88 |  | 25.5 | 75 |  | 15.8 | 46 |
| 50 | 0 | 0 | 50 |  | 52.8 | 106 |  | 37.2 | 74 |  | 28.8 | 58 |  | 26.7 | 53 |

Supplementary Table 4: Quantification results obtained for meat extract mixtures. DNA isolates from meat extract mixtures consisting of sika deer (50%, 25%, 10% or 5%) and pig were used for calibration. Analyses were performed in duplicates.

| **Content** | | | |  | **Calibrator 50% sika deer** | |  | **Calibrator 25% sika deer** | |  | **Calibrator 10% sika deer** | |  | **Calibrator 5% sika deer** | |
| --- | --- | --- | --- | --- | --- | --- | --- | --- | --- | --- | --- | --- | --- | --- | --- |
| **Sika deer  (%)** | **Red deer  (%)** | **Fallow deer  (%)** | **Pig  (%)** |  | **Mean sika deer content determined (%)** | **Mean recovery (%)** |  | **Mean sika deer content determined (%)** | **Mean recovery (%)** |  | **Mean sika deer content determined (%)** | **Mean recovery (%)** |  | **Mean sika deer content determined (%)** | **Mean recovery (%)** |
| **Meat extract mixtures** | | | | | | | | | | | | | | | |
| 1 | 0 | 0 | 99 |  | 1.9 | 187 |  | 2.0 | 198 |  | 1.3 | 128 |  | 1.4 | 137 |
| 1 | 1 | 1 | 97 |  | 1.7 | 172 |  | 2.1 | 215 |  | 1.4 | 138 |  | 1.4 | 137 |
| 1 | 19 | 19 | 61 |  | 1.2 | 122 |  | 1.5 | 148 |  | 1.1 | 109 |  | 1.2 | 115 |
| 1 | 0 | 1 | 98 |  | 2.1 | 208 |  | 2.4 | 240 |  | 1.8 | 184 |  | 1.7 | 170 |
| 2 | 0 | 0 | 98 |  | 2.2 | 111 |  | 2.8 | 142 |  | 2.0 | 102 |  | 2.1 | 106 |
| 2 | 2 | 0 | 96 |  | 2.6 | 131 |  | 3.0 | 148 |  | 1.8 | 92 |  | 2.2 | 112 |
| 2 | 2 | 2 | 94 |  | 2.0 | 102 |  | 2.1 | 105 |  | 1.6 | 82 |  | 1.7 | 83 |
| 2 | 38 | 0 | 60 |  | 1.8 | 91 |  | 2.3 | 113 |  | 1.6 | 78 |  | 1.6 | 80 |
| 2 | 0 | 38 | 60 |  | 1.2 | 59 |  | 1.6 | 81 |  | 0.8 | 41 |  | 1.1 | 56 |
| 2 | 19 | 19 | 60 |  | 1.8 | 91 |  | 1.9 | 97 |  | 1.2 | 60 |  | 1.4 | 68 |
| 5 | 33 | 0 | 62 |  | 5.1 | 101 |  | 5.2 | 104 |  | 3.9 | 79 |  | 4.5 | 91 |
| 5 | 15 | 15 | 65 |  | 4.4 | 88 |  | 3.7 | 74 |  | 3.3 | 67 |  | 3.5 | 69 |
| 5 | 0 | 0 | 95 |  | 6.6 | 132 |  | 6.2 | 124 |  | 5.3 | 106 |  | 5.7 | 114 |
| 5 | 5 | 0 | 90 |  | 5.5 | 110 |  | 5.9 | 118 |  | 4.6 | 91 |  | 4.8 | 95 |
| 5 | 5 | 5 | 85 |  | 5.3 | 106 |  | 5.4 | 107 |  | 4.3 | 87 |  | 4.5 | 91 |
| 5 | 0 | 33 | 62 |  | 4.1 | 82 |  | 4.3 | 85 |  | 3.1 | 63 |  | 4.8 | 96 |
| 10 | 0 | 0 | 90 |  | 11.7 | 117 |  | 11.5 | 115 |  | 9.4 | 94 |  | 9.4 | 94 |
| 10 | 10 | 0 | 80 |  | 10.4 | 104 |  | 11.1 | 111 |  | 9.2 | 92 |  | 10.0 | 100 |
| 10 | 10 | 10 | 70 |  | 8.1 | 81 |  | 9.7 | 97 |  | 9.1 | 91 |  | 8.4 | 84 |
| 20 | 0 | 0 | 80 |  | 20.8 | 104 |  | 22.0 | 110 |  | 18.4 | 92 |  | 19.0 | 95 |
| 20 | 20 | 0 | 60 |  | 21.2 | 106 |  | 20.4 | 102 |  | 18.1 | 90 |  | 18.1 | 91 |
| 20 | 0 | 20 | 60 |  | 14.4 | 72 |  | 15.4 | 77 |  | 13.6 | 68 |  | 13.4 | 67 |
| 20 | 20 | 20 | 40 |  | 15.0 | 75 |  | 14.6 | 73 |  | 14.1 | 71 |  | 11.8 | 59 |
| 25 | 0 | 0 | 75 |  | 28.2 | 113 |  | 26.7 | 107 |  | 24.8 | 99 |  | 23.5 | 94 |
| 25 | 25 | 0 | 50 |  | 24.0 | 96 |  | 25.0 | 100 |  | 23.2 | 93 |  | 21.0 | 84 |
| 25 | 25 | 25 | 25 |  | 13.7 | 55 |  | 13.8 | 55 |  | 12.9 | 52 |  | 12.3 | 49 |
| 33 | 0 | 0 | 67 |  | 37.3 | 113 |  | 38.6 | 117 |  | 34.1 | 103 |  | 30.2 | 92 |
| 33 | 5 | 0 | 62 |  | 35.4 | 107 |  | 35.4 | 107 |  | 28.2 | 85 |  | 27.8 | 84 |
| 33 | 5 | 5 | 57 |  | 30.4 | 92 |  | 29.2 | 89 |  | 25.8 | 78 |  | 26.3 | 80 |
| 38 | 0 | 0 | 62 |  | 39.6 | 104 |  | 38.7 | 102 |  | 33.5 | 88 |  | 33.1 | 87 |
| 38 | 5 | 5 | 52 |  | 32.4 | 85 |  | 34.3 | 90 |  | 29.4 | 77 |  | 29.4 | 77 |
| 34 | 33 | 33 | 0 |  | 15.1 | 44 |  | 16.6 | 49 |  | 15.8 | 46 |  | 13.9 | 41 |
| 50 | 0 | 0 | 50 |  | 49.1 | 98 |  | 51.4 | 103 |  | 48.7 | 97 |  | 46.1 | 92 |

Supplementary Table 5: Quantification results obtained for meat mixtures. DNA isolates from meat mixtures consisting of sika deer (50%, 28%, 10% or 5%) and pig were used for calibration. Analyses were performed in duplicates.

| **Content** | | | |  | **Calibrator 50% sika deer** | |  | **Calibrator 28% sika deer** | |  | **Calibrator 10% sika deer** | |  | **Calibrator 5% sika deer** | |
| --- | --- | --- | --- | --- | --- | --- | --- | --- | --- | --- | --- | --- | --- | --- | --- |
| **Sika deer  (%)** | **Red deer  (%)** | **Fallow deer (%)** | **Pig  (%)** |  | **Mean sika deer content determined (%)** | **Mean recovery (%)** |  | **Mean sika deer content determined (%)** | **Mean recovery (%)** |  | **Mean sika deer content determined (%)** | **Mean recovery (%)** |  | **Mean sika deer content determined (%)** | **Mean recovery (%)** |
| **Meat mixtures** | | | | | | | | | | | | | | | |
| 1 | 0 | 0 | 99 |  | 1.7 | 163 |  | 1.9 | 188 |  | 1.4 | 142 |  | 1.2 | 114 |
| 1 | 1 | 1 | 96 |  | 1.8 | 182 |  | 1.5 | 144 |  | 1.7 | 164 |  | 1.4 | 134 |
| 1 | 20 | 19 | 60 |  | 1.4 | 102 |  | 1.3 | 90 |  | 1.1 | 82 |  | 0.9 | 66 |
| 1 | 0 | 1 | 98 |  | 2.0 | 211 |  | 1.6 | 164 |  | 1.2 | 129 |  | 0.8 | 86 |
| 2 | 0 | 0 | 98 |  | 3.5 | 180 |  | 2.7 | 135 |  | 2.5 | 128 |  | 1.9 | 95 |
| 2 | 2 | 0 | 96 |  | 3.0 | 157 |  | 2.8 | 146 |  | 2.3 | 119 |  | 2.0 | 106 |
| 2 | 2 | 2 | 94 |  | 3.1 | 146 |  | 2.5 | 119 |  | 3.0 | 142 |  | 1.9 | 91 |
| 2 | 38 | 0 | 60 |  | 2.5 | 128 |  | 1.9 | 96 |  | 1.7 | 90 |  | 1.7 | 86 |
| 2 | 0 | 38 | 59 |  | 1.6 | 79 |  | 1.3 | 63 |  | 1.5 | 73 |  | 0.9 | 44 |
| 2 | 20 | 19 | 59 |  | 2.1 | 101 |  | 1.9 | 91 |  | 1.5 | 74 |  | 1.5 | 71 |
| 5 | 32 | 0 | 63 |  | 6.2 | 122 |  | 5.0 | 99 |  | 4.5 | 90 |  | 3.2 | 64 |
| 5 | 16 | 15 | 64 |  | 5.7 | 117 |  | 4.8 | 99 |  | 4.2 | 86 |  | 4.5 | 91 |
| 5 | 0 | 0 | 95 |  | 8.0 | 153 |  | 7.4 | 142 |  | 5.5 | 105 |  | 5.6 | 106 |
| 5 | 5 | 0 | 90 |  | 7.1 | 137 |  | 5.8 | 113 |  | 4.9 | 95 |  | 3.3 | 64 |
| 5 | 5 | 6 | 83 |  | 8.0 | 146 |  | 8.4 | 154 |  | 6.4 | 118 |  | 6.2 | 112 |
| 5 | 0 | 33 | 62 |  | 4.9 | 96 |  | 3.9 | 77 |  | 2.3 | 46 |  | 2.6 | 52 |
| 10 | 0 | 0 | 90 |  | 14.7 | 142 |  | 13.7 | 132 |  | 10.4 | 100 |  | 10.1 | 97 |
| 11 | 10 | 0 | 79 |  | 13.3 | 125 |  | 10.8 | 102 |  | 9.5 | 89 |  | 8.8 | 83 |
| 13 | 10 | 10 | 67 |  | 12.3 | 96 |  | 10.7 | 84 |  | 10.5 | 83 |  | 9.0 | 71 |
| 20 | 0 | 0 | 80 |  | 25.4 | 126 |  | 21.0 | 104 |  | 19.7 | 97 |  | 17.5 | 86 |
| 21 | 20 | 0 | 60 |  | 21.3 | 104 |  | 18.7 | 91 |  | 18.2 | 89 |  | 17.5 | 85 |
| 20 | 0 | 20 | 60 |  | 15.5 | 77 |  | 12.6 | 62 |  | 11.4 | 56 |  | 13.3 | 66 |
| 21 | 20 | 21 | 38 |  | 14.9 | 72 |  | 13.4 | 65 |  | 11.8 | 57 |  | 12.3 | 59 |
| 28 | 0 | 0 | 72 |  | 33.5 | 122 |  | 27.8 | 101 |  | 29.0 | 105 |  | 24.7 | 90 |
| 25 | 26 | 0 | 49 |  | 25.0 | 101 |  | 18.7 | 75 |  | 19.5 | 79 |  | 20.5 | 83 |
| 25 | 25 | 25 | 25 |  | 17.0 | 68 |  | 13.4 | 53 |  | 14.0 | 56 |  | 13.0 | 52 |
| 34 | 0 | 0 | 66 |  | 37.9 | 111 |  | 30.7 | 90 |  | 27.7 | 81 |  | 27.6 | 81 |
| 33 | 6 | 0 | 61 |  | 33.8 | 104 |  | 27.7 | 85 |  | 24.4 | 75 |  | 25.5 | 78 |
| 32 | 5 | 6 | 57 |  | 29.1 | 90 |  | 24.3 | 76 |  | 30.5 | 95 |  | 25.6 | 80 |
| 38 | 0 | 0 | 62 |  | 39.8 | 105 |  | 32.6 | 86 |  | 26.8 | 71 |  | 32.1 | 85 |
| 38 | 5 | 6 | 51 |  | 32.9 | 86 |  | 27.6 | 72 |  | 26.1 | 68 |  | 24.9 | 65 |
| 32 | 33 | 35 | 0 |  | 15.4 | 47 |  | 13.4 | 41 |  | 13.7 | 42 |  | 14.7 | 45 |
| 50 | 0 | 0 | 50 |  | 56.8 | 113 |  | 46.0 | 92 |  | 40.6 | 81 |  | 37.4 | 75 |
